# Assessment of exercise-induced stress via automated measurement of salivary cortisol concentrations and the testosterone-to-cortisol ratio: a preliminary study

**DOI:** 10.1038/s41598-023-41620-5

**Published:** 2023-09-04

**Authors:** Katsuhiko Tsunekawa, Yoshifumi Shoho, Kazumi Ushiki, Yoshimaro Yanagawa, Ryutaro Matsumoto, Nozomi Shimoda, Tomoyuki Aoki, Akihiro Yoshida, Kiyomi Nakajima, Takao Kimura, Masami Murakami

**Affiliations:** 1 Department of Clinical Laboratory Medicine, Gunma University Graduate School of Medicine, 3-39-22 Showa-machi, Maebashi, Gunma 371-8511 Japan; 2 Faculty of Education, Ikuei University, 1656-1 Kyome-machi, Takasaki, Gunma 370-0011 Japan

**Keywords:** Steroid hormones, Diagnostic markers

## Abstract

In this study, our aim was to validate whether the automated measurement of salivary testosterone and cortisol concentrations and the testosterone-to-cortisol (T/C) ratio, considering their individual circadian rhythms can be used to assess the stress response of male athletes to different exercise intensities accurately and effectively. We measured the salivary testosterone and cortisol concentrations and their respective serum concentrations that were collected from 20 male long-distance runners via passive drooling in the morning and evening for two consecutive days involving different exercise intensities. An electrochemiluminescence immunoassay was performed to evaluate the salivary testosterone and cortisol concentrations. The results showed a positive correlation between the salivary testosterone and cortisol concentrations and their respective serum concentrations. The participants were divided into two groups: with and without interval training. The interval training group showed a significantly higher rate of change in the salivary cortisol concentration and a significantly lower rate of change in the T/C ratio in the evening interval training on day 1 than lower-intensity running on day 2. Our results indicated that the salivary cortisol concentrations and the T/C ratio could distinguish between exercises at different intensities, which may be beneficial for detecting differences in stress responses among athletes.

## Introduction

In men, physical and psychological stresses caused by different factors, including resistance and endurance exercises, increase the secretion of cortisol and testosterone, which in turn affect the hypothalamic pituitary axis^1^. These hormones have their own circadian rhythms: the circulating cortisol concentration peaks 30 min after wakeup and then immediately decreases toward the evening, and the circulating testosterone concentration peaks at wakeup and gradually decreases toward the evening^1,2^. Overtraining syndrome refers to the hormonal response to increased physical and psychological stresses on athletes caused by excessive overloading during training, which results in reduced performance^3^. Cortisol shows a reduced response to exercise and changes in its circadian rhythm, such as a low resting concentration and peak loss after wakeup^3,4^. Testosterone also shows a decrease in its resting concentration^4^. Monitoring hormone secretions and their circadian rhythms can help with preventing and assessing overtraining syndrome. The testosterone-to-cortisol (T/C) ratio has also been shown to decrease with exercise-induced stress^5^. Testosterone and cortisol have their own circadian rhythms. Thus, the individual circadian rhythms of testosterone, cortisol, and the T/C ratio should be considered when monitoring exercise-induced stress among athletes.

Cortisol and testosterone concentrations are commonly assessed by using immunological methods on serum and saliva specimens. Saliva sampling is a simple, stress-free, and noninvasive method that does not require the help of a medical professional, such as for blood sampling^6^. Moreover, cortisol and testosterone concentrations are commonly conjugated to corticosteroid-binding globulin and sex hormone-binding globulin, respectively, in serum. However, both steroid hormones are not conjugated in saliva. Therefore, salivary cortisol and testosterone concentrations have stronger positive correlations with serum free cortisol and testosterone concentrations than with serum total cortisol and testosterone concentrations, respectively^7,8^. However, measuring the cortisol and testosterone concentrations to monitor stress caused by different exercise intensities must consider their individual circadian rhythms. This requires sequential sampling during days of exercise and the efficient measurement of a large number of samples. Our previous study showed that automated electrochemiluminescence immunoassay (ECLIA) can accurately assess the salivary cortisol concentrations, and sequential saliva sampling and automated measurement of salivary cortisol can be used to detect the circadian rhythm and compare stresses induced by endurance exercises of different intensities among female long-distance runners at the same time on different days^9,10^. Escribano et al.^11^ reported that an automated chemiluminescent immunoassay accurately evaluated the salivary testosterone concentrations of growing pigs. However, there have been no studies on similar saliva sampling and automated assessment of the testosterone concentration and T/C ratio among human athletes for assessing exercise stress. If the combined automated assessment of salivary testosterone and cortisol concentrations considering their individual circadian rhythms can adequately identify changes in stress caused by exercises at different intensities, this approach may help with establishing exercise programs that prevent overtraining syndrome. In the current study, our aim was to validate whether the automated ECLIA-based assessment of testosterone and cortisol concentrations and the T/C ratio can assess the stress response of male long-distance runners to exercises of varying intensities accurately and effectively.

## Methods

### Ethical approval and consent to participate

Written informed consent was obtained from all participants. This study was approved by Gunma University ethics review board for medical research involving human subjects, and registered with University Hospital Medical Information Network Clinical Trials Registry (UMIN-CTR) which meets the criteria of international committee of medical journal editors (UMIN registration number UMIN000051749, UMIN000051750). All measurements were carried out by trained athletes and in accordance with the Declaration of Helsinki.

### Participants

**Figure 1:**
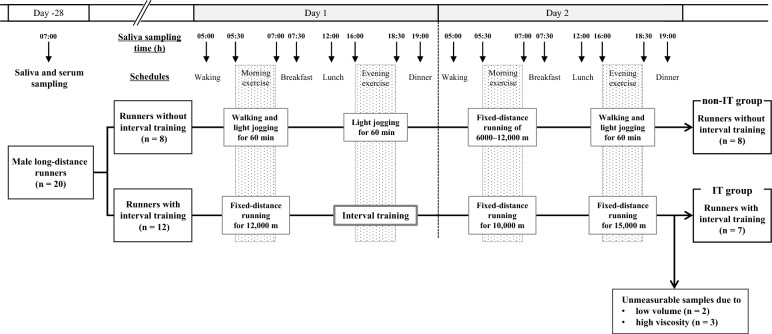
The study design and the division of male long-distance runners into two groups. The downwards arrows present the time of saliva sampling from the runners. non-IT group, non-interval training group; IT group, interval training group.

We recruited 20 elite Japanese male long-distance runners as participants. Their lifestyle habits (e.g., wakeup time, mealtime, bedtime, and meal contents) were standardized at the same dormitory. The study design is shown in Fig. 1. For the correlation analyses of the testosterone and cortisol concentrations, saliva and serum samples were collected at 7:00 am before their morning exercise and breakfast. After 28 days, saliva samples were collected sequentially from the participants in the morning and evening for two consecutive days, during which they performed exercises of different types and intensities. This took place during a training period sufficiently removed from races and followed the same procedure as described in previous studies^9,10^. Then, we divided the participants into two groups, which were defined as with and without interval training, respectively, in the evening on day 1. The non-interval training (non-IT) group was given the following exercise program. On day 1, they performed walking and light jogging for 60 min in the morning, and they performed light jogging for 60 min in the evening. On day 2, they performed fixed-distance running of 6000–12,000 m according to their individual conditions in the morning, and they performed walking and light jogging for 60 min in the evening. The interval training (IT) group was given the following exercise program. On day 1, they performed fixed-distance running for 12,000 m in the morning, and they performed interval training with seven sets of fast running for 1000 m and light jogging in the evening. On day 2, they performed fixed-distance running for 10,000 m in the morning, and they performed fixed-distance running for 15,000 m in the evening. The participants ran at their own pace during the light jogging, fixed-distance running, and interval training. To load faster running within a short time period, interval training was used for grouping as high-intensity exercise program. The participants were provided sufficient drinking water during training sessions to prevent dehydration^9,10^. On the two training days, saliva samples were collected at eight time points: upon waking (5:00 am), before morning exercise (5:30 am), after morning exercise (7:00 am), before breakfast (7:30 am), before lunch (12:00 pm), before evening exercise (16:00 pm), after evening exercise (18:30 pm), and before dinner (19:00 pm). This followed the same schedule as described in previous studies^9,10^.

### Physical examination and assessment of exercise intensity

The body weight and fat of the participants were assessed by using a bioimpedance instrument (InBody 770; InBody Japan, Tokyo, Japan). The body mass index was calculated as the weight in kilograms divided by the squared height in meters (kg/m^2^). Participants were interviewed on their use of medications and supplements. The participants wore Fitbit Ionic (Fitbit Inc. Tokyo, Japan), which is a reliable tool for measuring distances and pulse rates^12^, on their wrists the day before saliva collection. The resting pulse rate at wakeup time and the maximum pulse rates during each exercise session were assessed by using the wearable devices. The distance and duration of running during each exercise session were recorded, and the running velocity was calculated as the distance divided by the duration (m/min) following the procedure described in previous studies^9,10^. The Borg rating of perceived exertion (RPE) scale^13^ was used to assess the athlete's subjective exertion after exercise. The runner's RPE was scored on a scale of 6 to 20 and analyzed as the Borg scale score^9,10^.

### Sample collection

Saliva samples (targeted at 500 µL) were collected by unstimulated passive drooling using a polypropylene tube (SaliCap, IBL International, Hamburg, Germany). The participants were not allowed to brush their teeth, chew gum, or consume any food or drink except water within the 15 min before sample collection. All saliva samples were immediately stored at − 80 °C until analysis. Blood samples (2 mL) were obtained by puncturing an antecubital vein using a 23-G needle while the participants were sitting. The serum samples were separated via centrifugation (1500 × *g*) at 4 °C for 10 min, and they were immediately stored at − 80 °C until analysis^9,10^.

### Assessment of salivary and serum testosterone and cortisol concentrations

ECLIA was performed to assess the salivary and serum testosterone and cortisol concentrations by using the Elecsys Testosterone II and Elecsys Cortisol II on the Cobas 8000 system (Roche Diagnostics K.K, Tokyo, Japan). The intra- and inter-assay coefficients of variation (CVs) were respectively 4.2% and 5.6% for the salivary testosterone concentration, 1.7% and 1.6% for the serum testosterone concentration, 4.1% and 4.6% for the salivary cortisol concentration, and 1.3% and 3.4% for the serum cortisol concentration. The serum free testosterone concentration was evaluated by using the radioimmunoassay kit (IMMUNOTECH s.r.o., Prague, Czech Republic) at SRL Inc. (Tokyo, Japan). The intra- and inter-assay CVs of the serum free testosterone concentration were 5.9% and 6.5%, respectively. The salivary T/C ratio was calculated as the salivary testosterone concentration divided by the salivary cortisol concentration. The serum total testosterone-to-total cortisol ratio was calculated as the serum total testosterone concentration divided by the serum total cortisol concentration, and the serum free testosterone-to-total cortisol ratio was calculated as the serum free testosterone concentration divided by the serum total cortisol concentration. The rates of change in the salivary testosterone and cortisol concentrations and the salivary T/C ratio caused by exercise were calculated as each concentration and ratio after exercise divided by the corresponding concentration and ratio before exercise (%).

### Statistical analysis

The variables did not have a normal distribution. Thus, the measurement results were expressed as the median value and corresponding 25th–75th percentile range rather than mean values with standard deviations. Spearman’s correlation analysis was performed to assess the correlations between the salivary and serum testosterone and cortisol concentrations and the T/C ratio. The Mann–Whitney *U* test was used to identify statistically significant differences in each variable between the two groups. The Wilcoxon signed-rank test was utilized to identify statistically significant differences in each variable between two different time points. A *p* value of < 0.05 was considered statistically significant. All statistical analyses were performed by using the program SPSS Statistics version 26.0 (IBM Corp., Armonk, NY, USA).

## Results

### Correlations between the salivary and serum testosterone and cortisol concentrations and the T/C ratio

**Figure 2:**
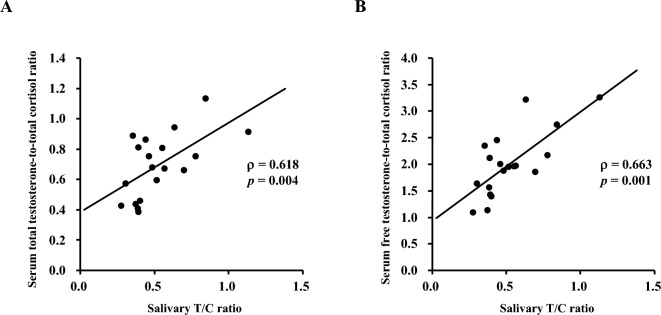
Spearman’s correlation analysis results for testosterone and cortisol concentrations in 20 male long-distance runners. (**A**) Between the salivary testosterone-to-cortisol ratio and the serum total testosterone-to-total cortisol ratio. (**B**) Between the salivary testosterone-to-cortisol ratio and serum free testosterone-to-total cortisol ratio.

Table 1 presents the characteristics of the participants. Table 2 shows the Spearman’s correlation analyses of the salivary and serum testosterone and cortisol concentrations obtained from ECLIA. The salivary testosterone concentration was positively correlated with the serum total testosterone concentration (ρ = 0.702, *p* < 0.001) and serum free testosterone concentration (ρ = 0.789, *p* < 0.001). The salivary cortisol concentration was positively correlated with the serum total cortisol concentration (ρ = 0.586, *p* = 0.007). The salivary T/C ratio was significantly positively correlated with the serum total testosterone-to-total cortisol ratio (ρ = 0.618, *p* = 0.004) and serum free testosterone-to-total cortisol ratio (ρ = 0.663, *p* = 0.001). Figure 2 shows the significant positive correlations between the salivary T/C ratio and the serum total testosterone-to-total cortisol ratio (Fig. 2A), and between the salivary T/C ratio and the serum free testosterone-to-total cortisol ratio (Fig. 2B).

**Table 1:** Characteristics of participants.

Characteristics	Value
Number	20
Age (years)	19.0 (19.0–19.0)
Height (cm)	170.5 (166.5–173.0)
Weight (kg)	57.1 (54.6–60.8)
Body mass index (kg/m^2^)	19.5 (19.1–20.0)
Body fat (%)	11.4 (9.3–13.3)

Data are expressed as the median (25th–75th percentile).

**Table 2:** Correlations between the salivary and serum testosterone and cortisol concentrations and the T/C ratio.

Saliva	Serum	ρ	*p* value
Testosterone concentration (ng/mL)	Total testosterone concentration (ng/mL)	0.702	< 0.001
	Free testosterone concentration (pg/mL)	0.789	< 0.001
Cortisol concentration (µg/dL)	Total cortisol concentration (µg/dL)	0.586	0.007
T/C ratio	Total testosterone-to-total cortisol ratio	0.618	0.004
	Free testosterone-to-total cortisol ratio	0.663	0.001

Correlations between the salivary and serum testosterone and cortisol concentrations and testosterone-to-cortisol (T/C) ratio were analyzed by Spearman’s test.

### Running intensity of each exercise program

Figure 1 shows the division of the participants into two groups. Eight runners without the interval training in evening exercise on day 1 were included in non-IT group, and 12 runners with interval training were included in IT group. Five runners in the IT group were excluded because measurable saliva samples could not be obtained owing to low volume (n = 2) and high viscosity (n = 3), which resulted in seven runners with measurable samples. Table 3 shows the characteristics of each group. There were no significant differences in terms of height, weight, body mass index, body fat, and resting pulse rate on days 1 and 2 between the groups. Table 4 presents the running intensity during each exercise program in each group. In the evening exercise on day 1, the IT group who did interval training had a significantly higher running velocity (*p* = 0.014), Borg scale score (*p* < 0.001), and maximum pulse rate (*p* = 0.001) than the non-IT group. In addition, the IT group had a significantly higher running velocity (*p*_*e*_ = 0.043), Borg scale score (*p*_*e*_ = 0.018), and maximum pulse rate (*p*_*e*_ = 0.018) during the evening exercise on day 1 than on day 2. The IT group had a significantly higher Borg scale score (*p*_*day1*_ = 0.017) on day 1 during the evening exercise than during the morning exercise. The IT group had a significantly higher running velocity (*p* = 0.043) during the morning exercise on day 1 than the non-IT group. The IT group had a significantly higher running velocity (*p*_*m*_ = 0.046) during the morning exercise on day 1 than the morning exercise on day 2. The IT group had a significantly longer running distance (*p* = 0.009) than the non-IT group during the evening exercise on day 2, but the running velocity, Borg scale score, and maximum pulse rate did not differ significantly. The IT group had a significantly longer running distance (*p*_*day2*_ = 0.028) during the evening exercise than during the morning exercise on day 2. The non-IT group had a significantly longer running distance (*p*_*day1*_ = 0.043) during the evening exercise than during the morning exercise on day 1. The non-IT group had a significantly higher running velocity (*p*_*m*_ = 0.043) and Borg scale score (*p*_*m*_ = 0.041) during the morning exercise on day 2 than on day 1.

**Table 3:** Characteristics of the two groups with or without interval training.

	Non-IT group	IT group	*p* value
Number	8	7	
Age (years)	19.0 (18.5–20.5)	19.0 (19.0–19.0)	0.694
Height (cm)	169.5 (168.0–171.0)	166.0 (165.5–172.5)	0.536
Weight (kg)	55.2 (53.7–57.2)	57.5 (53.9–59.8)	0.536
Body mass index (kg/m^2^)	19.1 (18.8–19.9)	19.6 (19.4–20.1)	0.232
Body fat (%)	11.2 (7.3–13.8)	11.6 (10.4–12.9)	0.779
Resting pulse rate on day 1 (beats/min)	55 (53–59)	48 (43–50.5)	0.121
Resting pulse rate on day 2 (beats/min)	59 (53–60)	47 (42–49)	0.072

Data are expressed as the median (25th–75th percentiles).

The participants were divided into two groups with and without the interval training, and the variables were compared using the Mann–Whitney *U* test.

Non-IT group, non-interval training group; IT group, interval training group.

**Table 4:** Running intensity during each exercise program.

	Group	Day 1			Day 2			Day 1 versus 2	
		Morning exercise	Evening exercise	*p*_*day1*_	Morning exercise	Evening exercise	*p*_*day2*_	*p*_*m*_	*p*_*e*_
Exercise program	Non-IT (n = 8)	Walking and jogging	Jogging		Fixed-distance running	Walking and jogging			
	IT (n = 7)	Fixed-distance running	Interval training		Fixed-distance running	Fixed-distance running			
Running distance (m)	Non-IT (n = 8)	7751 (6527–10,490)	9420 (7225–11,500)	0.043	11,000 (6850–12,000)	9215 (8000–13,500)	0.496	0.075	0.441
	IT (n = 7)	12,000 (10,935–12,000)	10,000 (9100–12,500)	0.866	10,270 (10,000–11,300)	15,070 (15,000–15,605)**	0.028	0.141	0.116
Running velocity (m/min)	Non-IT (n = 8)	139.4 (127.5–167.3)	157.0 (141.7–177.4)	0.116	200.0 (136.7–217.5)	157.8 (111.6–177.8)	0.123	0.043	1.000
	IT (n = 7)	200.0 (179.2–236.8)*	268.8 (211.8–314.8)*	0.176	166.7 (163.6–188.4)	180.7 (175.7–207.2)	0.398	0.046	0.043
Borg scale score	Non-IT (n = 8)	10.0 (8.0–13.0)	11.0 (8.5–11.0)	0.932	12.5 (11.5–14.0)	11.0 (9.5–11.5)	0.175	0.041	0.893
	IT (n = 7)	12.0 (11.0–13.0)	16.0 (15.5–17.5)**	0.017	11.0 (9.5–11.5)	12.0 (9.5–13.5)	0.078	0.063	0.018
Maximum pulse rate (beats/min)	Non-IT (n = 8)	169 (151–175)	160 (138–173)	0.262	172 (163–183)	136 (120–170)	0.068	0.176	0.225
	IT (n = 7)	181 (177–187)	196 (194–200)**	0.063	173 (137–175)	144 (142–179)	0.612	0.046	0.018

Data are expressed as the median (25th–75th percentiles).

*p < 0.05 and **p < 0.01 for variables between patients classified into two groups with and without the interval training in the evening on day 1 using the Mann–Whitney *U* test.

*p*_day1_ morning exercise versus evening exercise on day 1 using the Wilcoxon signed-rank test.

*p*_day2_ morning exercise versus evening exercise on day 2 using the Wilcoxon signed-rank test.

*p*_m_ day 1 versus 2 for morning exercise using the Wilcoxon signed-rank test.

*p*_e_ day 1 versus 2 for evening exercise using the Wilcoxon signed-rank test.

Non-IT, non-interval training; IT, interval training.

### Changes in the salivary testosterone and cortisol concentrations and the T/C ratio in response to exercise intensity

**Figure 3:**
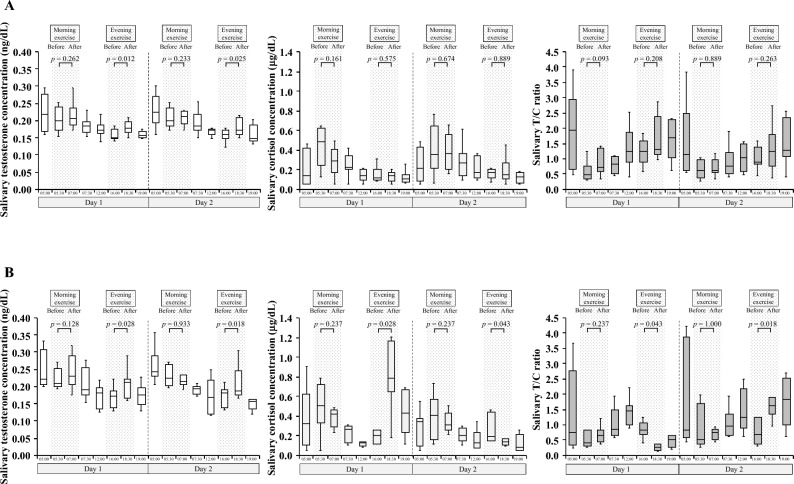
Changes in the salivary testosterone concentration, salivary cortisol concentration, and salivary testosterone-to-cortisol ratio over two consecutive days in response to each exercise program and considering their circadian rhythms. (**A**) non-interval training group (n = 8). (**B**) interval training group (n = 7).

Figure 3 depicts the changes in the salivary testosterone and cortisol concentrations and the T/C ratio in response to exercise and considering the circadian rhythms of the two groups over two consecutive days. There was no significant difference between the two groups in the testosterone concentration at wakeup on day 1 (*p* = 0.336) and day 2 (*p* = 0.397). There was also no significant difference between the two groups in the cortisol concentration at wakeup on day 1 (*p* = 0.232) and day 2 (*p* = 0.867). There was no significant difference between the two groups in the T/C ratio at wakeup on day 1 (*p* = 0.463) and day 2 (*p* = 1.000). The salivary testosterone concentration gradually decreased from morning to evening, and the salivary cortisol concentration peaked after wakeup (5:30 am) and immediately decreased on both days for the two groups. Furthermore, the T/C ratio reached its minimum after wakeup (5:30 am) and then gradually increased. Changes in the salivary testosterone and cortisol concentrations and T/C ratio during their individual circadian rhythms were detected on both days for the two groups. The salivary testosterone and cortisol concentrations and the T/C ratio did not show significant changes after the morning exercise. However, the salivary testosterone concentration significantly increased after the evening exercise on both days for both groups. The non-IT group did not show significant changes in the salivary cortisol concentration and T/C ratio after the evening exercise on both days. However, the IT group showed a significant increase in the salivary cortisol concentration after the evening exercise on day 1 and a significant decrease on day 2. The IT group also showed a significant decrease in the T/C ratio after the evening exercise on day 1 and a significant increase on day 2.

### Rates of change in the salivary testosterone and cortisol concentrations and the T/C ratio

**Figure 4:**
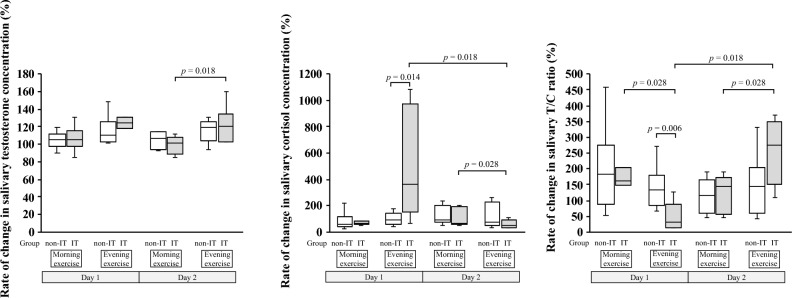
Rates of change in the salivary testosterone and cortisol concentrations and the T/C ratio caused by each exercise in the two groups. The white box plots present the rate of changes in each parameter in the non-interval training (non-IT) group, and the gray box plots indicate those in the interval training (IT) group. The differences between the two groups were analyzed by using the Mann–Whitney *U* test. The differences between two time points of exercise among participants in the same group were analyzed by using the Wilcoxon signed-rank test.

Figure 4 shows the rates of change in the salivary testosterone and cortisol concentrations and the T/C ratio. According to the Wilcoxon signed-rank test, the IT group showed a significantly higher rate of change in the salivary cortisol concentration (*p* = 0.018) and a significantly lower rate of change in the T/C ratio (*p* = 0.018) during the evening exercise on day 1 than on day 2. On day 1, the IT group showed a significantly lower rate of change in the T/C ratio (*p* = 0.028) in the evening than in the morning. On day 2, the IT group showed significantly higher rates of change in the salivary testosterone concentration (*p* = 0.018) and T/C ratio (*p* = 0.028) and a significantly lower rate of change in the salivary cortisol concentration (*p* = 0.028) during the evening exercise than during the morning exercise. According to the Mann–Whitney *U* test, the IT group had a significantly higher rate of change in the salivary cortisol concentration (*p* = 0.014) and a significantly lower rate of change in the T/C ratio (*p* = 0.006) than the non-IT group during the evening exercise on day 1.

## Discussion

In this study, we investigated whether the automated measurement of the salivary testosterone and cortisol concentrations and the salivary T/C ratio can be used to assess the stress induced by exercise at different intensities among male long-distance runners accurately and effectively while considering circadian rhythms. The salivary testosterone and cortisol concentrations showed positive correlations with their respective serum concentrations. The combination of sequential saliva collection and automated ECLIA measurement was able to detect the circadian rhythms of the testosterone and cortisol concentrations and the T/C ratio, as well as acute changes caused by exercise. However, measurable saliva samples could not be obtained via passive drooling from five participants due to low volume and high viscosity. The IT group showed a significantly higher rate of change in the salivary cortisol concentration and a significantly lower rate of change in the salivary T/C ratio during interval training in the evening on day 1, as well as the multiple intensity indices. Such changes were not observed in the salivary testosterone concentration.

The cotton swab is a convenient method for quickly collecting a sufficient volume of saliva without residue and mucus for assessing stress marker levels^14^. The cortisol concentration in cotton swab samples is a better predictor of the serum total cortisol and free cortisol concentrations than passive drooling among healthy volunteers^7^. In contrast, cotton swab samples have falsely high testosterone concentrations when assessed by immunoassays^15,16^. Previous studies have shown that the cross-reactivity between plant hormones and antibodies influence the results of the testosterone immunoassay if a cotton swab is used^15–18^. This is why we used passive drooling in the current study. The salivary cortisol concentration measured by automated second-generation ECLIA has a significantly positive correlation with the measurement by liquid chromatography–tandem mass spectrometry (LC–MS/MS)^19^. We previously assessed the exercise-induced stress among female long-distance runners by combining automated measurement of the salivary cortisol concentration with sequential sampling using a cotton swab^9,10^. In that study, the salivary cortisol concentration from ECLIA showed a significantly positive correlation with the concentration from an enzyme-linked immunosorbent assay^9^. Moreover, all salivary samples collected by a cotton swab could be measured without some needing to be excluded due to a low dose or high viscosity samples^9,10^.

In the present study, we first evaluated the salivary testosterone concentration using ECLIA by comparing them to the serum samples. The salivary testosterone concentration showed a significantly more positive correlation with the serum free testosterone concentration than with the serum total testosterone concentration. The salivary testosterone concentration can be combined with sequential sampling using passive drooling to assess the circadian rhythm among runners. However, the saliva samples of several participants that were collected via passive drooling did not have sufficient volume and were extremely viscous, so they could not be utilized for ECLIA. Physical and mental stresses reduce the flow rate and increase the viscosity of saliva^20,21^. Assays including ECLIA require a sufficient sample volume (minimum of 100–200 µL) because of the dead volume required for trouble-free automated sample processing. Automated ECLIA for measuring the salivary cortisol and testosterone concentrations is advantageous because it can measure a large number of samples easily and rapidly. However, its usefulness may be reduced if used in combination with passive drooling owing to its requirement for large sample volumes without residue and mucus.

Previous studies have shown that the serum cortisol concentration acutely increases due to moderate- to high-intensity endurance exercise (i.e., > 60% of the maximal oxygen consumption [VO_2 max_])^22,23^. Sato et al.^24^ showed that the serum cortisol and free testosterone concentrations in healthy young men were elevated by two 15-min sessions of submaximal exercise using an electromechanically braked ergometer at ≥ 40% of their peak oxygen uptake (VO_2 peak_) among non-athletes. However, the serum cortisol and free testosterone concentrations were only increased among male endurance runners by exercise at 90% VO_2 peak_. Tremblay et al.^25^ showed that a running duration of at least 80 min increases testosterone and cortisol concentrations during low-intensity endurance exercise. Resistance exercise acutely increases testosterone secretion, which is an anabolic hormone that is essential for muscular adaptation and muscle growth^1^. In a previous meta-analysis, Hayes et al.^5^ revealed that although acute aerobic and resistance exercises consistently increase the salivary testosterone concentration, the acute response of salivary testosterone to power-based exercise has not been fully elucidated. Anderson et al.^26^ showed that the serum free testosterone concentration decreases immediately after exhaustive endurance exercise and gradually increases after 24 h or during the recovery process among male endurance athletes.

In the current study, the interval training during the evening on day 1 for the IT group had the highest exercise intensity based on indicators including the running velocity, Borg scale score, and maximum pulse rate. The interval training significantly increased the salivary cortisol concentration and decreased the salivary T/C ratio. The rate of change in the salivary testosterone concentration showed no significant differences for a given exercise between the two groups or between different exercises within the same group. The salivary testosterone concentration increased after the evening exercise on both days for both groups, despite the differences in exercise intensity. The circadian rhythm of serum testosterone is characterized by high concentrations in the morning followed by a gradual decline in the evening, accompanied by a mild rise from 16:00 to 19:00 pm in young men^27^. In our study, the increase in salivary testosterone concentrations after evening exercise at 18:30 pm in all runners, independent of exercise intensity, may have detected this circadian rhythm. Moreover, the change in the salivary testosterone concentration may have been affected by a longer exercise duration. Doan et al.^28^ observed similar results in the circadian rhythm during a 36-hole golf competition, in which the salivary testosterone concentration only increased during holes 25–30 in the evening on the competition day compared with the baseline day. In contrast, the salivary cortisol concentration increased and the salivary T/C ratio decreased at almost every hole on the competition day. They concluded that a low T/C ratio was correlated with good golf performance. The T/C ratio is generally an indicator of the anabolic/catabolic balance during skeletal muscle destruction and recovery^29^. In the current study, we observed that a lower salivary T/C ratio might reflect the acute stress response to exercises of different intensities. However, we did not assess differences in the performance of participants or changes in hormone levels during recovery. Thus, further study is need on the salivary testosterone concentration and T/C ratio to investigate their associations with the performance and recovery processes of endurance- and resistance-trained athletes.

Detecting the responses of testosterone and cortisol to exercise in the morning is a challenge owing to their respective circadian rhythms. However, such measurements are easily obtained in the evening^30^. We^9^ previously showed that differences in the rate of change in the salivary cortisol concentration caused by exercise at different intensities could be compared at the same time on different days, even in the early morning. This method allowed us to assess the differences in acclimatization and exercise stress between two altitudes^10^. In the current study, the rates of change in the salivary testosterone and cortisol concentrations and the T/C ratio caused by morning exercise showed no differences between each group on both days. This may be because the exercise intensity was not sufficient to elicit a hormonal response in this study compared with the exercises used in our previous studies^9,10^. However, we did observe significant differences in the running velocity and maximum pulse rate in the morning. Further studies involving other types of exercises and higher intensities must be conducted to compare the changes in the hormonal response to exercise in the early morning. In contrast, the rates of change in the cortisol concentration and T/C ratio caused by evening exercise showed significant differences between the two days depending on the exercise intensity. This result suggests that automated salivary cortisol assessment to compare exercise-induced stress response in our previous studies is also useful in determining the salivary T/C ratio. Based on its circadian rhythm, the T/C ratio was lowest after wakeup and then gradually increased. We believe this reflects the circadian rhythm of cortisol rather than that of testosterone. The rate of change in the salivary T/C ratio indicated differences in the stress response between each exercise program. The salivary T/C ratio decreased on day 1 and increased on day 2 after the evening exercise for the IT group. We concluded that it was more influenced by the cortisol response than the testosterone response. In their meta-analysis, Hayes et al.^5^ obtained similar results showing that the response of the salivary T/C ratio to exercise was due to changes in the salivary cortisol concentration. Because passive drooling may not be sufficient for obtaining the samples required for assessing the T/C ratio, further study should be conducted to evaluate whether cortisol alone can be used to evaluate the exercise-induced stress response. This would be very helpful because cortisol can be measured by using saliva conventionally collected with cotton swabs.

The current study had several limitations. First, the sample size of each group was relatively small. Furthermore, the number of participants in the IT group was reduced because some saliva samples obtained via passive drooling could not be used for the automated measurement. In addition, we were unable to compare the circadian rhythms between sedentary participants without exercise effect and the exercised runners. The reason for this limitation was the focus on standardizing living conditions that prevented the recruitment of large numbers of well-trained runners or non-runners. Moreover, it was not possible to provide a sedentary period because maintaining the condition of the runners was top priority. Second, the exercise programs were not evaluated by using accurate intensity indicators such as VO_2 max_. The runner’s exercise conditions could not be tightly controlled. In contrast, we formed two groups with or without high-intensity interval training in which multiple intensity indices were significantly higher, and we consider it important to be able to assess the difference in stress response by salivary cortisol concentration and T/C ratio between higher-intensity interval training and lower-intensity running on different days only in IT group. Further studies are needed to set the sedentary group showing the circadian rhythms without exercise effect, and to use a standardized exercise program with more participants and accurate indicators for both the high- and low-intensity exercise. Third, the time between the post-evening exercise and pre-dinner sampling points was not sufficient. Some participants had high salivary hormone concentrations at the pre-dinner sampling point, which should be when they are lowest particularly for cortisol. More studies should be performed to adjust the collection time according to the training program, such as including a point before bedtime to assess the basal concentrations at night.

In conclusion, automated ECLIA assessment of salivary testosterone and cortisol concentrations is as accurate as an assessment using serum samples. The cortisol concentration and T/C ratio assessed via sequential saliva collection and automated evaluations can adequately reflect differences in endurance exercise intensity on different days performed at the same time. Such an approach may be useful for detecting different stress responses among athletes while considering the circadian rhythm.

## Data Availability

The datasets used and/or analyzed during the current study are available from the corresponding author on reasonable request.

## References

[1] Kraemer WJ, Ratamess NA (2005). Hormonal responses and adaptations to resistance exercise and training. Sports Med..

[2] Clow A, Hucklebridge F, Stalder T, Evans P, Thorn L (2010). The cortisol awakening response: More than a measure of HPA axis function. Neurosci. Biobehav. Rev..

[3] Urhausen A, Kindermann W (2002). Diagnosis of overtraining: What tools do we have?. Sports Med..

[4] Cadegiani FA, Kater CE (2019). Novel causes and consequences of overtraining syndrome: The EROS-DISRUPTORS study. BMC Sports Sci. Med. Rehabil..

[5] Hayes LD, Grace FM, Baker JS, Sculthorpe N (2015). Exercise-induced responses in salivary testosterone, cortisol, and their ratios in men: A meta-analysis. Sports Med..

[6] Hofman LF (2001). Human saliva as a diagnostic specimen. J. Nutr..

[7] Poll EM (2007). Saliva collection method affects predictability of serum cortisol. Clin. Chim. Acta.

[8] Lippi G (2016). Analytical evaluation of free testosterone and cortisol immunoassays in saliva as a reliable alternative to serum in sports medicine. J. Clin. Lab. Anal..

[9] Ushiki K (2020). Assessment of exercise-induced stress by automated measurement of salivary cortisol concentrations within the circadian rhythm in Japanese female long-distance runners. Sports Med. Open.

[10] Tsunekawa K (2022). Differences in stress response between two altitudes assessed by salivary cortisol levels within circadian rhythms in long-distance runners. Sci. Rep..

[11] Escribano D, Fuentes-Rubio M, Cerón JJ (2014). Salivary testosterone measurements in growing pigs: Validation of an automated chemiluminescent immunoassay and its possible use as an acute stress marker. Res. Vet. Sci..

[12] Fuller D (2021). Predicting lying, sitting, walking and running using Apple Watch and Fitbit data. BMJ Open Sport Exerc. Med..

[13] Borg GAV (1982). Psychophysical bases of perceived exertion. Med. Sci. Sports Exerc..

[14] Granger DA (2007). Integration of salivary biomarkers into developmental and behaviorally oriented research: Problems and solutions for collecting specimens. Physiol. Behav..

[15] Shirtcliff EA, Granger DA, Schwartz E, Curran MJ (2001). Use of salivary biomarkers in biobehavioral research: Cotton-based sample collection methods can interfere with salivary immunoassay results. Psychoneuroendocrinology.

[16] Büttler RM (2018). Testosterone, androstenedione, cortisol and cortisone levels in human unstimulated, stimulated and parotid saliva. Steroids.

[17] Dabbs JM (1991). Salivary testosterone measurements: Collecting, storing, and mailing saliva samples. Physiol. Behav..

[18] Granger DA, Schwartz EB, Booth A, Arentz M (1999). Salivary testosterone determination in studies of child health and development. Horm. Behav..

[19] Gagnon N (2018). Establishment of reference intervals for the salivary cortisol circadian cycle, by electrochemiluminescence (ECLIA), in healthy adults. Clin. Biochem..

[20] Matos-Gomes N (2010). Psychological stress and its influence on salivary flow rate, total protein concentration and IgA, IgG and IgM titers. NeuroImmunoModulation.

[21] Ligtenberg AJM, Liem EH, Brand HS, Veerman EC (2016). The effect of exercise on salivary viscosity. Diagnostics (Basel).

[22] Davies CTM, Few JD (1973). Effects of exercise on adrenocortical function. J. Appl. Physiol..

[23] Hill EE (2008). Exercise and circulating cortisol levels: The intensity threshold effect. J. Endocrinol. Invest..

[24] Sato K (2016). Responses of sex steroid hormones to different intensities of exercise in endurance athletes. Exp. Physiol..

[25] Tremblay MS, Copeland JL, Van Helder W (2005). Influence of exercise duration on post-exercise steroid hormone responses in trained males. Eur. J. Appl. Physiol..

[26] Anderson T, Lane AR, Hackney AC (2016). Cortisol and testosterone dynamics following exhaustive endurance exercise. Eur. J. Appl. Physiol..

[27] Bremner WJ, Vitiello MV, Prinz PN (1983). Loss of circadian rhythmicity in blood testosterone levels with aging in normal men. J. Clin. Endocrinol. Metab..

[28] Doan BK, Newton RU, Kraemer WJ, Kwon YH, Scheet TP (2007). Salivary cortisol, testosterone, and T/C ratio responses during a 36-hole golf competition. Int. J. Sports Med..

[29] Gatti R, De Palo EF (2011). An update: salivary hormones and physical exercise. Scand. J. Med. Sci. Sports.

[30] Chtourou H (2014). The effect of time of day on hormonal responses to resistance exercise. Biol. Rhythm Res..

