# Author Correction: Assessment of exercise-induced stress via automated measurement of salivary cortisol concentrations and the testosterone-to-cortisol ratio: a preliminary study

**DOI:** 10.1038/s41598-024-51789-y

**Published:** 2024-01-15

**Authors:** Katsuhiko Tsunekawa, Yoshifumi Shoho, Kazumi Ushiki, Yoshimaro Yanagawa, Ryutaro Matsumoto, Nozomi Shimoda, Tomoyuki Aoki, Akihiro Yoshida, Kiyomi Nakajima, Takao Kimura, Masami Murakami

**Affiliations:** 1 Department of Clinical Laboratory Medicine, Gunma University Graduate School of Medicine, 3-39-22 Showa-Machi, Maebashi, Gunma 371-8511 Japan; 2 Faculty of Education, Ikuei University, 1656-1 Kyome-Machi, Takasaki, Gunma 370-0011 Japan

Correction to: *Scientific Reports* 10.1038/s41598-023-41620-5, published online 04 September 2023

The Acknowledgments section in the original version of this Article was incomplete.

“We are grateful to Hidekazu Shimazu, Koji Hase, Mai Murata, Larasati Martha, and Mayumi Nishiyama for providing technical assistance and helpful discussion. This work was supported by the Ministry of Education, Culture, Sports, Science, and Technology of Japan [grant numbers 18K07406 (K. Tsunekawa)].”

now reads:

“We are grateful to Hidekazu Shimazu, Koji Hase, Mai Murata, Larasati Martha, and Mayumi Nishiyama for providing technical assistance and helpful discussion. This work was supported by the Ministry of Education, Culture, Sports, Science, and Technology of Japan [grant numbers 18K07406 and 23K10581 (K. Tsunekawa)].”

The original Article has been corrected.

